# The association between sedentary behaviors during weekdays and weekend with change in body composition in young adults

**DOI:** 10.3934/publichealth.2016.2.375

**Published:** 2016-06-03

**Authors:** Clemens Drenowatz, Madison M. DeMello, Robin P. Shook, Gregory A. Hand, Stephanie Burgess, Steven N. Blair

**Affiliations:** 1 Department of Exercise Science, University of South Carolina, Columbia, SC; 2 Department of Kinesiology, Iowa State University, Ames, IA; 3 Department of Epidemiology, West Virginia University, Morgantown, WV; 4 College of Nursing, University of South Carolina, Columbia, SC; 5 Department of Epidemiology and Biostatistics University of South Carolina, Columbia, SC

**Keywords:** body weight, body fat, sedentary time, TV time, computer time, sitting time, physical activity

## Abstract

**Background:**

High sedentary time has been considered an important chronic disease risk factor but there is only limited information on the association of specific sedentary behaviors on weekdays and weekend-days with body composition. The present study examines the prospective association of total sedentary time and specific sedentary behaviors during weekdays and the weekend with body composition in young adults.

**Methods:**

A total of 332 adults (50% male; 27.7 ± 3.7 years) were followed over a period of 1 year. Time spent sedentary, excluding sleep (SED), and in physical activity (PA) during weekdays and weekend-days was objectively assessed every 3 months with a multi-sensor device over a period of at least 8 days. In addition, participants reported sitting time, TV time and non-work related time spent at the computer separately for weekdays and the weekend. Fat mass and fat free mass were assessed via dual x-ray absorptiometry and used to calculate percent body fat (%BF). Energy intake was estimated based on TDEE and change in body composition.

**Results:**

Cross-sectional analyses showed a significant correlation between SED and body composition (0.18 ≤ *r* ≤ 0.34). Associations between body weight and specific sedentary behaviors were less pronounced and significant during weekdays only (*r* ≤ 0.16). Nevertheless, decrease in SED during weekends, rather than during weekdays, was significantly associated with subsequent decrease in %BF (*β* = 0.06, *p* <0.01). After adjusting for PA and energy intake, results for SED were no longer significant. Only the association between change in sitting time during weekends and subsequent %BF was independent from change in PA or energy intake (β_%BF_ = 0.04, *p* = 0.01), while there was no significant association between TV or computer time and subsequent body composition.

**Conclusions:**

The stronger prospective association between sedentary behavior during weekends with subsequent body composition emphasizes the importance of leisure time behavior in weight management.

## 1. Introduction

Excess sedentary time has been associated with various adverse health outcomes including cardiovascular disease, diabetes, metabolic syndrome, certain types of cancer and mortality [1]–[4]. Sedentary behaviors are defined as activities performed while seated or lying down with an energy expenditure of less than 1.5 METs (metabolic equivalent) during waking hours [5]. Given the changes in modes of transportation, communications, workplace and domestic physical activity demands due to economic advances and industrial innovations, a reduction in time spent sedentary has become a key area for public health separately from the promotion of physical activity [1], [6]–[8]. Sedentary behaviors have also been shown to have distinctly different physiological effects from PA [9]. Accordingly, sedentary behavior should be no longer considered simply as the absence of moderate-to-vigorous physical activity (MVPA) but rather as a specific set of behaviors, characterized by low energy requirements and unique health consequences [10].

High sedentary time has also been associated with excess body weight but there remains controversy on the directionality of this association [11]. Results from the Nurses' Health Study indicate that high sedentary time, particularly watching TV, is associated with a higher risk for excess weight gain [12]. Other studies, however, indicate that increased body weight is a precursor rather than a consequence of high sedentary time in adults [13], [14]. The lack of conclusive evidence may, in part, be due to variability in the assessment of sedentary time. Most studies relied on self-reported behaviors, such as TV time, as a proxy measure for total daily sedentary time [15]. Such measures, however, only capture leisure time sedentary behavior. Even though TV time is a prominent sedentary behavior there is limited information on the association between TV time and objectively determined total sedentary time, particularly during working days. Further, a change in media consumption due to the availability of mobile devices such as smart phones and tablets may contribute to a greater variability in screen time that may not be captured by focusing only on TV time. More research, therefore, is needed to enhance our understanding of the contribution of specific sedentary behaviors on various health outcomes, including obesity [8]. In addition, there is a need to examine whether patterns of sedentary behaviors affect body composition differently. Specifically, differences in behavioral choices during weekdays and weekend-days may affect body composition differently as weight gain has been shown to be more pronounced during the weekend compared to weekdays [16]. The purpose of the present study was to examine the prospective association between objectively measured sedentary time as well as self-reported sedentary behaviors during weekdays and the weekend and body composition.

## 2. Materials and Methods

The present study uses baseline through one-year follow-up data from a prospective, observational study, which has been described in detail elsewhere [17]. A total of 430 healthy young adults (49% male) between 20 and 35 years of age were recruited via e-mail listservs, website postings, posters and flyers in an urban area in South Carolina between July 2011 and February 2013. The study was designed to examine the interaction of physical activity, dietary behavior and body composition in young adults as this population is at particular risk for weight gain [18], [19]. Power analyses revealed that at least 300 participants are needed to detect an average weight gain of 1 kg/year with an effect size of 0.2. Inclusion criteria consisted of a BMI between 19 and 35 kg/m^2^, no major chronic or acute health conditions and no major changes in health behaviors in the three months prior to entering the study. Pregnant women, women who were planning on getting pregnant and those planning to change their use of contraceptive medications in the following 2 years were also excluded. The study protocol was approved by the University of South Carolina Institutional Review Board and all participants signed an informed consent prior to data collection.

Anthropometric measurements were obtained every three months by trained and certified research staff. Body weight (kg) and height (cm) were measured with participants in surgical scrubs and bare feet to the nearest 0.1 kg and 0.1 cm, respectively. Body mass index (BMI, kg/m^2^) was calculated using the average of 3 measures obtained at each measurement visit. Baseline measures were used to classify participants as normal weight (BMI < 25 kg/m^2^) or overweight/obese (BMI ≥ 25 kg/m^2^) Total fat mass (FM) and fat free mass (FFM) were assessed via dual X-ray absorptiometry (DXA, Lunar DPX^®^ system, version 3.6; Lunar Radiation Corp, Madison, WI) and percent body fat (BF) was calculated (FM/body weight). Due to the limitations of diet reports [20], energy intake (EI) was calculated based on change in FM and FFM and average total daily energy expenditure (TDEE) for each respective 3-month period [21], [22].

TDEE, time spent sedentary and in moderate-to-vigorous physical activity (MVPA) were determined using the SenseWear Mini Armband (Body Media, Pittsburgh, PA). The armband is worn on the upper arm and combines measurements of near body temperature, skin temperature, heat flux, galvanic skin response, and accelerometry. Several studies have examined the validity of the armband in various populations and previous research indicated accurate estimates of energy expenditure, physical activity (PA) and sleep in free-living adults [23]–[26]. Participants were asked to wear the armband for 24 hours/day and only remove it during periods when it might get wet (e.g., taking a shower or swimming). Participants were given the armband for a period of up to 14 days every 3 months. Compliance was set at 7 days of wear-time, including Saturday and Sunday, with a minimum of 18 hours of wear-time per day. Data for Saturday and Sunday was averaged to reflect weekend PA and the average from Monday through Friday was used to reflect weekday PA. SenseWear's propriatory algorithm (version 7.0 professional) estimates energy expenditure minute-by-minute and subsequently determines TDEE as well as time spent sedentary, excluding sleep (sedentary < 1.5 METs), time spent in light PA (1.5 METs ≤ LPA < 3 METs), and time spent in MVPA (MVPA ≥ 6 METs).

During periods of non-wear time participants reported their activities. Using individual resting metabolic rate (RMR), energy expenditure during non-weartime was estimated based on the Compendium of PA [27]. RMR was measured via indirect calorimetry (True One 2400, Parvo Medics, Sandy, UT) after a 12-hour overnight fast and 24 hour abstention from exercise in a dimly lit room for 45 minutes. RMR was determined as the average of 10 consecutive minutes with the lowest coefficient of variation.

Information on specific sedentary behaviors was obtained via self-report. Specifically, participants were asked about time spent sitting, watching TV and non-work related computer use during a typical weekday and weekend-day. Average time spent in various sedentary behaviors for the entire week was calculated using a 5:2 ratio for weekdays and weekend-days.

*Statistical Analysis.* In order to be included in the analysis, valid data needed to be available for at least 3 measurement time points, including baseline and 12-month follow-up. Upon confirmation of normal distribution of the data differences between compliant and non-compliant participants were examined via ANOVA and Chi-square tests for categorical variables. Differences between weekdays and weekend were examined via paired-sample t-tests. Correlation analyses were used to examine the cross-sectional association between objectively determined sedentary time, self-reported sedentary behavior and body composition. The strength of the association was defined as weak (0.1 > *r* > 0.3), moderate (0.3 ≥ *r* ≥ 0.5) or strong (*r* > 0.5) for positive and negative trends [28]. Changes in body composition, sedentary time and specific sedentary behaviors were determined via linear mixed modeling. Subsequently, the association between change in sedentary behavior and body composition at 1-year follow-up was examined via linear regression analysis, adjusting for age, baseline sedentary time and baseline body composition; a second model also included time spent in MVPA and calculated EI. Analyses were carried out for the total sample and separately for normal weight and overweight/obese participants due to potential differences in the association between sedentary behavior and body composition by weight category [29]. All analyses were conducted using IBM SPSS Statistics for Windows (version 22.0; IBM Corp., Armonk, NY).

## 3. Results

A total of 332 adults (49.7% male) provided valid data throughout the 1-year observation period. Two thirds (66.6%) of the sample were white/Caucasian with a majority (86%) having a college degree. Baseline descriptive characteristics did not differ between compliant and non-compliant participants, except for a higher prevalence of college graduates in compliant compared to non-compliant participants (86.4% vs. 74.5%, *p* = 0.01). Average armband wear-time for compliant participants was 23.1 ± 0.6 hours/day for a total of 50.2 ± 3.3 days over the one-year observation period. Table 1 shows baseline measures for all compliant participants and separately for normal weight and overweight/obese participants. There were no significant differences in sex distribution, education or ethnicity between weight categories. Further, men and women did not differ in time spent sedentary or specific sedentary behaviors even though men displayed more time spent in MVPA. In addition to the expected differences in body composition, normal weight participants were younger than overweight/obese (*p* = 0.01). Participants spent 66.2 ± 8.5% of their waking hours in sedentary pursuits. Normal weight participants displayed lower sedentary time and more time in MVPA (*p* < 0.01). Sedentary time during waking hours was lower during the weekend compared to weekdays (*p* < 0.01) due to longer sleep duration; there was no difference in light PA and MVPA between weekdays and the weekend. Self-reported time spent at specific sedentary behaviors did not differ between normal weight and overweight obese participants. Sitting time and time spent at the computer was significantly higher on weekdays compared to weekends (*p* ≤ 0.01). TV time, on the other hand, was significantly higher on weekends compared to weekdays (*p* < 0.01).

Correlations between total sedentary time and time spent at specific sedentary behaviors were generally weak. Objectively determined sedentary time was significantly correlated with sitting time during weekdays and weekends. The correlation between objectively measured sedentary time and TV time as well as time spent at the computer, however, was only significant during weekends (Table 2). Correlations between time spent in specific sedentary behaviors and time spent in MVPA were weak as well, even though total sedentary time was inversely correlated with time spent in MVPA (*r* < -0.6). The correlations between weekday and weekend behaviors were strong for objective measurements, moderate for sitting time but only weak for computer time and TV time (Table 3). Correlations for change in weekend and weekday behaviors throughout the 12-month observation period were generally weak, except for change in MVPA (*r* = 0.4) and change in sitting time (*r* = 0.3). Accordingly, change in objectively determined sedentary time was not significantly correlated with change in specific sedentary behaviors.

Cross-sectional analyses between body composition and sedentary time showed a low to moderate association between body composition and total sedentary time, particularly in overweight/obese (Table 4). The correlation was stronger during weekdays compared to weekend sedentary time. Associations with specific behaviors were less pronounced and only significant in normal weight participants. Specifically, sitting time and TV time during weekdays were directly correlated with %BF while weekend computer time was directly correlated with BMI.

Over the 1-year observation period, participants experienced a weight gain of 1.0 ± 3.4 kg, which was associated with an increase of 0.7 ± 2.5 %BF. Weight change did not differ between normal weight and overweight groups, but change in %BF was more pronounced in those who were normal weight at baseline. Change in objectively determined sedentary time and specific sedentary behavior did not differ between normal weight and overweight/obese participants.

Regression analyses did not show a significant prospective association between change in sedentary time and body weight at 12-month follow-up after adjusting for baseline body weight (Table 5). Baseline sedentary time, however, was associated with body composition at 1-year follow-up, particularly in overweight/obese. Further, an increase in sedentary time during the weekend was associated with higher %BF at follow-up. This result, however, was no longer significant after adjusting for change in MVPA and energy intake. There was no significant association between change in specific sedentary behaviors and subsequent body composition, except for the association between change in sitting time during weekends and %BF in overweight/obese (*β* = 0.04; *p* = 0.01). This association remained after additionally adjusting for MVPA and EI.

There was also an association between change in body weight and total sedentary time at 1-year follow-up in overweight/obese participants (*β* = 0.13; *p* = 0.04). No significant associations were observed between change in body composition and specific self-reported sedentary behaviors at 1-year follow-up. Analyzing men and women separately revealed similar results as those shown for the total sample.

**Table 1.** Descriptive characteristics at Baseline. for the total sample and separately for Normal Weight and Overweight/obese participants. Values are Mean ± SD.

	Total Sample (N = 332)		Normal Weight (N = 183)		Overweight/Obese (N = 149)	
Age (yrs)	27.7 ± 3.7		27.2 ± 3.5		28.3 ± 3.9	
Height (kg)	171.9 ± 9.6		171.8 ± 9.6		172.0 ± 9.5	
Weight (kg)	74.6 ± 13.8		66.3 ± 9.3		84.7 ± 11.3	
BMI (kg/m^2^)	25.2 ± 3.8		22.4 ± 1.6		28.6 ± 2.8	
Fat Mass (kg)	21.2 ± 8.6		16.4 ± 4.9		27.0 ± 8.6	
Fat Free Mass (kg)	54.0 ± 11.2		50.4 ± 10.6		58.1 ± 11.0	
% Body Fat	28.3 ± 9.1		25.2 ± 7.8		32.0 ± 9.2	
	Weekdays	Weekends	Weekdays	Weekends	Weekdays	Weekends
MVPA (min/day)	136.6 ± 79.5	136.7 ± 96.5	165.0 ± 78.8	166.1 ± 101.4	101.7 ± 65.4	100.6 ± 76.0
Sed. excl. Sleep (min/day)	698.5 ± 102.0	649.5 ± 119.6	674.7 ± 93.1	624.4 ± 113.2	727.7 ± 105.1	680.2 ± 9.9
Sitting time (min/day)	433.7 ± 209.1	366.2 ± 198.1	436.5 ± 202.2	367.3 ± 192.3	430.2 ± 218.0	365.7 ± 206.1
TV time (min/day)	158.4 ± 94.8	193.0 ± 138.6	150.5 ± 94.1	191.0 ± 135.3	168.2 ± 95.0	195.5 ± 142.9
Non-work Comp.time (min/day)	201.1 ± 122.6	177.9 ± 132.8	195.1 ± 113.5	184.6 ± 136.8	208.4 ± 127.3	169.6 ± 127.8

Sed. excl. sleep… objectively determined sedentary time, excluding sleep; Comp.time… non-work related time spent at the computer

**Table 2.** Associations between objectively determined time spent sedentary or in MVPA and self-reported sedentary behaviors during weekdays and weekends at baseline. Values are Pearson correlation coefficients.

			Sitting^b^ (min/day)	TV time^b^ (min/day)	Computer^b^ (min/day)
**TOTAL SAMPLE**	***Weekdays***	Sedentary (min/day) ^a^	0.212 **	0.011	−0.013
		MVPA (min/day)	−0.129 *	−0.092	−0.080
	***Weekend***	Sedentary (min/day) ^a^	0.199 **	0.229 **	0.244 **
		MVPA (min/day)	−0.148 **	−0.018	−0.055
**NORMAL WEIGHT**	***Weekdays***	Sedentary (min/day) ^a^	0.210 **	−0.006	0.036
		MVPA (min/day)	−0.156 *	−0.062	−0.088
	***Weekend***	Sedentary (min/day) ^a^	0.228 **	0.125	0.258 **
		MVPA (min/day)	−0.158 *	0.026	−0.083
**OVERW./OBESE**	***Weekdays***	Sedentary (min/day) ^a^	0.238 **	−0.022	−0.090
		MVPA (min/day)	−0.137	−0.057	−0.031
	***Weekend***	Sedentary (min/day) ^a^	0.180 *	0.348 **	0.275 **
		MVPA (min/day)	−0.166 *	−0.076	−0.072

^a^ objectively assessed sedentary time excluding sleep; ^b^ self-reported sedentary time

MVPA… time spent in moderate-to-vigorous physical activity

* significant coefficient at *p* < 0.05 ** significant coefficient at *p* < 0.01

**Table 3.** Association between weekday and weekend behaviors at baseline. Values are Pearson correlation coefficients.

	MVPA (min/day)	Sedentary (min/day) ^a^	Sitting^b^ (min/day)	TV time^b^ (min/day)	Computer^b^ (min/day)
**TOTAL SAMPLE**	0.707 **	0.561 **	0.437 **	0.089	0.116 *
**NORMAL WEIGHT**	0.653 **	0.487 **	0.391 **	0.106	0.094
**OVERW./OBESE**	0.686 **	0.581 **	0.487 **	0.066	0.150

^a^ objectively assessed sedentary time excluding sleep; ^b^ self-reported sedentary time

MVPA… time spent in moderate-to-vigorous physical activity

* significant coefficient at *p* < 0.05 ** significant coefficient at *p* < 0.01

**Table 4.** Association between body composition and sedentary behavior during weekdays and the weekend at baseline. Values are Pearson correlation coefficients.

			Sedentary ^a^ (min/day)	Sitting^b^ (min/day)	TV time^b^ (min/day)	Computer^b^ (min/day)
***TOTAL SAMPLE***	***Week-days***	BMI(kg/m^2^)	0.340 **	0.006	0.079	0.085
		% Body Fat	0.298 **	0.128 *	0.157 **	0.095
	***Week-end***	BMI(kg/m^2^)	0.274 **	−0.002	0.034	−0.018
		% Body Fat	0.183 **	0.080	−0.028	−0.019
**NORMAL WEIGHT**	***Week-days***	BMI(kg/m^2^)	0.120	0.019	0.012	0.120
		% Body Fat	0.219 **	0.178 *	0.169 *	0.070
	***Week-end***	BMI(kg/m^2^)	0.111	−0.005	0.099	0.175 *
		% Body Fat	0.122	0.173 *	0.061	0.139
**OVERW./OBESE**	***Week-days***	BMI(kg/m^2^)	0.307 **	0.041	0.000	0.040
		% Body Fat	0.231 **	0.109	0.093	0.094
	***Week-end***	BMI(kg/m^2^)	0.183 *	0.003	−0.006	−0.043
		% Body Fat	0.094	0.001	−0.134	0.152

^a^ objectively assessed sedentary time excluding sleep; ^b^ self-reported sedentary time

* significant coefficient at *p* < 0.05 ** significant coefficient at *p* < 0.01

## 4. Discussion

The inverse cross-sectional association between body composition and sedentary behaviors has been shown previously [30], [31]. Results of the present study generally confirm previous findings but the strength of the associations were moderate at best with more pronounced results in overweight/obese compared to normal weight adults. Further, associations between body composition and self-reported specific behaviors (i.e. TV and computer time) were less pronounced than the association with total sedentary time. Thus, health promotion efforts should emphasize a reduction in total sedentary time, rather than specific sedentary behaviors. The main focus of the present study, however, was on the prospective association between sedentary behaviors and body composition and potential differences in the contribution of weekday and weekend behaviors. Similar to the cross-sectional results, associations between change in specific sedentary behaviors and subsequent body composition were limited. A review on the prospective association between sedentary behaviors and various health outcomes also concluded that evidence on a longitudinal relationship between sedentary behavior and body weight is limited [15]. In the present study, only sitting time during weekends was associated with subsequent body composition independent of PA. These results could provide viable information for intervention strategies as they highlight the importance of leisure time behavior in weight management. Of additional interest is that change in body weight was associated with higher subsequent sedentary time, which is consistent with previous research [13], [14], [32]. Taken together, there appears to be a bi-directional association between body composition and sedentary behavior in which weight gain and sedentary behavior are mutually reinforcing [15].

**Table 5.** Prospective associations between objectively measured changes in total sedentary time, excluding sleep, during weekdays and weekends and body composition at 1-year follow-up. Values are standardized regression coefficients ( ).

		Model	Baseline Sedentary time (min/day)	Δ Sedentary weekday (min/day)	Δ Sedentary weekend (min/day)	Δ MVPA (min/day)	Δ EI (kcal/day)
**TOTAL SAMPLE**	**BMI at 1-year follow-up (kg/m^2^)**	1	0.056 **	0.008	0.029	N / A	N / A
		2	0.067 **	−0.018	−0.005	−0.140 **	0.094 **
	**% Body Fat at 1-year follow-up**	1	0.060 *	0.020	0.063 **	N / A	N / A
		2	0.070 **	−0.018	0.031	−0.118 **	0.027
**NORMAL WEIGHT**	**BMI at 1-year follow-up (kg/m^2^)**	1	−0.006	−0.018	0.010	N / A	N / A
		2	0.030	−0.074	−0.075	−0.293 **	0.183 **
	**% Body Fat at 1-year follow-up**	1	0.017	0.006	0.062 *	N / A	N / A
		2	0.031	−0.047	0.012	−0.162 **	0.037
**OVERW./OBESE**	**BMI at 1-year follow-up (kg/m^2^)**	1	0.153 **	0.046	0.061	N / A	N / A
		2	0.159 **	0.008	0.031	−0.171 **	0.119 **
	**% Body Fat at 1-year follow-up**	1	0.112 **	0.048	0.064 **	N / A	N / A
		2	0.121 **	0.024	0.045	−0.084 **	0.026

* significant coefficient at *p* < 0.05 ** significant coefficient at *p* < 0.01

Δ… change in objectively determined sedentary behavior over 12 months

MVPA… moderate-to-vigorous physical activity over entire week;

EI… calculated energy intake

N/A indicates that variables were not included in the regression model

Models 1 and 2 are additionally adjusted for sex, age, and baseline body composition

A bi-directional association could also help with explaining differences in the association between weekday and weekend sedentary time with body composition. A higher body weight potentially induces a higher amount of sedentary time during weekdays when opportunities for light PA and MVPA are generally limited. The influence on occupational demands on total sedentary time is further indicated by the limited association between total sedentary time and specific behavioral choices (i.e. TV and computer time) during weekdays. The greater freedom for conscious choices during weekends, on the other hand, provides more options for a reduction in sedentary time by engaging in various forms of recreational activities of light intensity or structured MVPA in a potentially conscious effort to control body weight. The fact that people tend to engage in more active behaviors during the morning hours [33], [34] also suggests that weekends provide valuable options for interventions targeting a reduction of sedentary time as there will be less interference with occupational demands during these times. The higher caloric intake, generally observed during weekends further emphasizes the need for behavioral adaptations, such as an increase in PA, during weekends in order to avoid weight gain [16], [35]–[37]. Nevertheless, higher sedentary time during working hours has been associated with higher sedentary time on non-workdays [6], [38], [39] and there appears to be no difference in sedentary time between workdays and non-workdays [40]. Thus, intervention strategies addressing a reduction in sedentary time should target occupational and leisure time behaviors. Results of the present study further emphasize the importance of MVPA in weight management. Despite the fact that high sedentary time has been associated with increased risk for cardiovascular disease, type 2 diabetes and all-cause mortality independent of MVPA [41]–[43], weight loss may require efforts that go beyond a reduction of sedentary time, most likely including alterations in diet and PA.

Of additional interest is the weak association between specific self-reported sedentary behaviors and body composition, which emphasizes the need for a comprehensive assessment of overall sedentariness when examining the health risks associated with excess sedentary time. Even though TV time has been commonly used as indicator for sedentary behavior, this behavior appears to contribute only a small proportion to total sedentary time, particularly in light of the high amount of occupational sedentary time [15]. Further, modern technology that provides access to online media via multiple outlets may contribute to shifts in sedentary behaviors, especially in young adults, that may not be captured by inquiries on single sedentary choices. Communicating on the phone, for example, has become one of the most commonly reported sedentary behaviors due to 24-hour access to mobile devices [44]. The simultaneous engagement in multiple sedentary behaviors may further contribute to the difficulty in accurately determining sedentary time based on specific behaviors. People may be talking on the phone while surfing the internet or performing other tasks such as posting messages on social media at the same time. In addition, there is the potential for bias with self-reported behaviors, which affects measurement accuracy and could have contributed to the limited significant findings between specific self-reported sedentary behaviors and body composition. There are, however, also some limitations with currently available objective measures of sedentary time due to the limited information on the content of specific behaviors. Given that TV viewing, for example, has been associated with a higher caloric intake [45], specific information on behavioral content may still be valuable in examining the role of sedentary behaviors in weight management. Further, most objective measurements base their classification of sedentary time predominantly on movement rather than body position. Thus, standing still may be classified as sedentary behavior even though sedentary behavior has been defined as activities performed in a seated or lying position [5].

Besides the concerns regarding measurement accuracy with either method additional limitations should be considered as well when interpreting the findings of this study. The present study did not provide information on the duration or number of bouts of sedentary pursuits. Interruptions of prolonged sedentary bouts, however, have been shown to affect various health outcomes, independent of total sedentary time [46]. Further, Saturday and Sunday was assumed to reflect non-work days for the entire sample. Given that the majority of participants were associated with a university, this is a plausible assumption but it may not reflect the actual work pattern of each individual participant. Nevertheless, Rowlands et al. argue that even in the absence of a full time job with a periodic work rhythm, people adjust their behavior to activities of others along with opening hours of various businesses, resulting in specific daily activity patterns that resembles a weekly pattern observed with employment [47]. It should also be considered that even though sedentary time was comparable to the US adult population, PA levels were higher in the study population [48], which may mitigate some of the detrimental effects of high sedentary time. The availability of repeated measurements throughout the observation period along with high compliance with the objective PA assessment, on the other hand, is a considerable strength of this study. Further, the inclusion of estimated energy intake and MVPA in the analyses provides additional information on the complex interaction of key contributors to weight management.

Overall, results of the present study emphasize the need for a comprehensive approach in weight management, including PA and diet. Even though the rise in occupational sedentary time has been of particular concern in modern society [6], [49], leisure time behavior appears to remain a crucial component in weight management. In fact occupational sedentary time has been shown to contribute less to total sedentary time than behavioral choices at home, particularly in young adults [44]. Despite the fact that the workplace has been identified as a target setting for health promotion [50], interventions targeting weight loss need to consider the importance of leisure time behavior in weight management. Accordingly, McCarthy argues for an increased awareness of the long-term consequences of short-lived weekend lifestyle choices and emphasizes the promotion of healthy behavioral choices during the weekend when people are generally facing a less structured daily routine [35]. The potential bi-directional association between body composition and sedentary time also highlights the need for preventive measures that help with limiting weight gain rather than focusing on weight loss in order to avoid a vicious cycle of excess weight and high sedentary time. A preventive approach, addressing behavior at work and during leisure time, also requires less severe behavioral changes and thus may allow for a better adherence to various intervention strategies.
